# Quantification and recognition of parkinsonian gait from monocular video imaging using kernel-based principal component analysis

**DOI:** 10.1186/1475-925X-10-99

**Published:** 2011-11-10

**Authors:** Shih-Wei Chen, Sheng-Huang Lin, Lun-De Liao, Hsin-Yi Lai, Yu-Cheng Pei, Te-Son Kuo, Chin-Teng Lin, Jyh-Yeong Chang, You-Yin Chen, Yu-Chun Lo, Shin-Yuan Chen, Robby Wu, Siny Tsang

**Affiliations:** 1 Department of Electrical Engineering, National Taiwan University, No. 1, Sec. 4, Roosevelt Rd., Taipei 106, Taiwan; 2 Department of Neurology, Tzu Chi General Hospital, Tzu Chi University, No. 707, Sec. 3, Chung, Yang Rd., Hualien 970, Taiwan; 3 Institute of Biomedical Engineering, National Taiwan University, No.1, Sec. 1, Jen-Ai Rd., Taipei 100, Taiwan; 4 Department of Electrical Engineering, National Chiao Tung University, No. 1001, Ta-Hsueh Rd., Hsinchu 300, Taiwan; 5 Department of Physical Medicine and Rehabilitation, Chang Gung Memorial Hospital at Linkou, No.5, Fusing St., Gueishan Township, Taoyuan County 333, Taiwan; 6 Brain Research Center, National Chiao Tung University, No. 1001, Ta-Hsueh Rd., Hsinchu 300, Taiwan; 7 Department of Biomedical Engineering, National Yang Ming University, No.155, Sec. 2, Linong St., Taipei 112, Taiwan; 8 Center for Optoelectronic Biomedicine, National Taiwan University College of Medicine, No. 1, Ren-Ai Rd., Sec. 1, Taipei, Taiwan; 9 Department of Neurosurgery, Tzu Chi General Hospital, Tzu Chi University, No. 707, Sec. 3, Chung Yang Rd., Hualien 970, Taiwan; 10 Philadelphia College of Osteopathic Medicine, 4170 City Avenue, Philadelphia, PA 19131, USA; 11 Department of Psychology, University of Virginia, No.102 Gilmer Hall, PO BOX 400400, Charlottesville, VA 22904-4400, USA

**Keywords:** Parkinson's disease, Kernel-based principal component analysis, power spectrum, classification, quantification

## Abstract

**Background:**

The computer-aided identification of specific gait patterns is an important issue in the assessment of Parkinson's disease (PD). In this study, a computer vision-based gait analysis approach is developed to assist the clinical assessments of PD with kernel-based principal component analysis (KPCA).

**Method:**

Twelve PD patients and twelve healthy adults with no neurological history or motor disorders within the past six months were recruited and separated according to their "Non-PD", "Drug-On", and "Drug-Off" states. The participants were asked to wear light-colored clothing and perform three walking trials through a corridor decorated with a navy curtain at their natural pace. The participants' gait performance during the steady-state walking period was captured by a digital camera for gait analysis. The collected walking image frames were then transformed into binary silhouettes for noise reduction and compression. Using the developed KPCA-based method, the features within the binary silhouettes can be extracted to quantitatively determine the gait cycle time, stride length, walking velocity, and cadence.

**Results and Discussion:**

The KPCA-based method uses a feature-extraction approach, which was verified to be more effective than traditional image area and principal component analysis (PCA) approaches in classifying "Non-PD" controls and "Drug-Off/On" PD patients. Encouragingly, this method has a high accuracy rate, 80.51%, for recognizing different gaits. Quantitative gait parameters are obtained, and the power spectrums of the patients' gaits are analyzed. We show that that the slow and irregular actions of PD patients during walking tend to transfer some of the power from the main lobe frequency to a lower frequency band. Our results indicate the feasibility of using gait performance to evaluate the motor function of patients with PD.

**Conclusion:**

This KPCA-based method requires only a digital camera and a decorated corridor setup. The ease of use and installation of the current method provides clinicians and researchers a low cost solution to monitor the progression of and the treatment to PD. In summary, the proposed method provides an alternative to perform gait analysis for patients with PD.

## Background

Parkinson's disease (PD) is a chronic degenerative disease [1]. Due to the absence of dopamine in the basal ganglia circuit in the brain, people with PD commonly present gait disorders that affect their walking ability and reduce the energy efficiency of their gait [2,3]. As gait disorders are symptoms of the early stage of PD, gait performance has become a specific and major hallmark in the assessment and evaluation of the disease's progression [4-6]. The Unified Parkinson's Disease Rating Scale (UPDRS) [7] is widely used to assess and track the longitudinal course of PD. Among UPDRS, part III is often used to evaluate the level of motor impairment and response to levodopa (L-dopa) for PD patients. However, such evaluations rely on the experience and/or the expertise of the clinician. Observations based on the part III of UPDRS tend to be subjective. In light of the issues mentioned, a quantitative measurement for parkinsonian gait is much needed.

In the past two decades, video-based motion analysis that requires no physical contact has become a popular solution for gait analysis [8,9]. Instead of traditional evaluations conducted by human inspectors, researchers now use computer-aided video gait analysis for precise data collection, reliable quantitative measurements, and systematic data management. As natural body movements can be transformed into essential spatial-temporal parameters with video-based motion analysis, abnormalities in gait and posture can be captured and identified with precision [10-14]. Conventional gait analysis is performed using an experimental setup consisting of force plates, multiple infrared cameras, reflective balls, light-emitting diodes (LED), or inertial sensors placed onto regions of the subject's body [15]. Although these systems provide accurate kinematic measurements, conventional gait analyses require a relatively large space and expensive equipment. Furthermore, dedicated manpower is required to calibrate the camera system, apply the reflective balls, and utilize the software. These properties of conventional gait analysis limit its application in many clinical settings, such as clinics with space or budgetary limitations.

However, gait analysis in PD patients is important for both determining the severity of PD and evaluating the improvements provided by the treatment regime. A method for performing gait analysis that can be applied in clinics within a limited space to discriminate between PD patients and healthy controls and to determine the therapeutic effect of L-dopa on PD patients is required. The silhouette method provides an economical alternative for recognizing and analyzing PD gait in a clinical setting [16]. The silhouette method records the silhouettes of the subjects walking and extracts the biometric features that are highly correlated with gait patterns using a single camera. Thus, the computational and storage requirements are largely reduced [17]. As lower-cost alternative methods that require less space and less dedicated manpower than the model-based method, the existing approaches that use walking silhouettes [18-21] perform well in recognizing different gaits but do not provide the quantitative measurements of the gait parameters used for monitoring abnormalities in or progression of parkinsonian gait.

In order to develop a simple and efficient method for the quantification and recognition of parkinsonian gait, a video-based silhouette approach using kernel-based principal component analysis (KPCA) [22] is developed in this study. Participant's gait performance during the steady-state walking period is captured and then analyzed to verify the proposed method. The aim of the approach is to provide clinicians and researchers with an easy-to-use and -install tool to recognize and quantify the gait performance of non-PD controls and PD patients in both "Drug-Off" and "Drug-On" states. Temporal and spectral analyses of gait patterns are applied to investigate the subjects' walking patterns.

## Methods

### The participants

Twelve PD patients who scored an average rating of 2.33 on the Hoehn and Yahr (H&Y) scale (six scored 2.5, five scored 2, and one scored 3) and twelve healthy adults with no neurological history that might cause motor disorders within the past six months were recruited from Hualien Buddhist Tzu Chi General Hospital, Taiwan. The subjects volunteered to participate, and informed consent was obtained from all subjects in accordance with Buddhist Tzu Chi General Hospital's Institutional Review Board (IRB 097-08) Committee on research involving human subjects. The biometric characteristics of the participants are listed in Table 1. The healthy adults were denoted as non-PD controls; the PD patients given L-dopa at an equivalent daily dose for one hour were classified as "Drug-On", whereas those that abstained from L-dopa treatment for at least 12 hours were classified as "Drug-Off". For the PD patients, the degree of motor function impairment was evaluated using part III of the UPDRS.

**Table 1:** The basic biometric characteristics of categorized subjects.

	**PD patients (9 M/3 F)**			**non-PD Controls (3 M/9 F)**		
		
	**Min**	**Max**	**Mean ± SD**	**Min**	**Max**	**Mean ± SD**
	
Age (years)	49.00	74.00	60.30 ± 6.71	48.00	67.00	56.40 ± 7.04
Height (m)	1.50	1.82	1.52 ± 0.46	1.49	1.80	1.60 ± 0.08
Weight (kg)	36.00	106.00	63.05 ± 24.37	50.00	67.00	58.08 ± 5.05
Body mass index (kg/m^2^)	13.38	36.25	24.90 ± 5.74	21.36	24.37	22.76 ± 1.15
Disease duration (years)	1.00	18.00	8.00 ± 4.82	N/A	N/A	N/A
Hoehn and Yahr stage	2	2.5	2.33 ± 0.33	N/A	N/A	N/A

SD = standard deviation

### Environmental setup and videotaping standard

A 6-m corridor decorated with a navy curtain was prepared for the walking trials. A commercial digital charge-coupled device (CCD) video camera (PV-GS400, Panasonic, Japan) was mounted on a tripod and placed 4.1 m in front of the curtain, perpendicular to the walking pathway, to capture the lateral view of each participant's walk.

All participants were asked to wear clothing of a much lighter color than the curtain to facilitate the filtration of noise during image post-processing. To reduce the variability of the gait performance, the participants were asked to perform three walking trials. All participants were asked to walk at their natural pace in order to naturally reflect their gait performance. Between trials, the participants were instructed to rest for at least five minutes until their strength was recovered.

The PD patients were asked to abstain from L-dopa overnight for at least 12 hours prior to the gait measurements. They then performed three drug-off trials in the morning. Immediately following the completion of the drug-off trials, they were given L-dopa at an equivalent daily dose. Three drug-on trials were then assessed one hour after the administration of L-dopa.

In this study, we utilized the participant's gait performance during the steady-state walking period to assist in the verification of the proposed method. Therefore, the participants were asked to begin walking 2 m from the left end of the corridor. Moreover, the last 1 m of each walking period was not videotaped so that patient deceleration did not affect the data.

**Figure 1:**
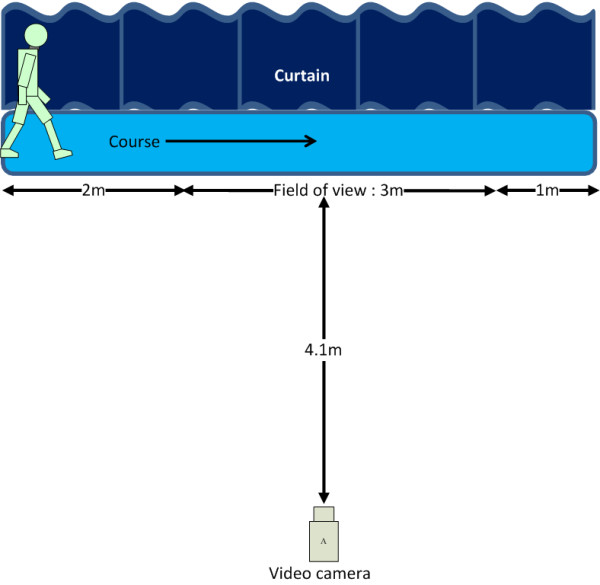
**A general schematic of the experimental setup used for video recording**. The participant wears a light suit to enhance the contrast between the individual and the dark background. The participant walks along the course (approximately 6 m) in front of the video camera (located approximately 4.1 m away). To ensure that the captured data reflect the gait performance during the steady-state walking period, the camera videotapes only the middle 3 m of each walking trial.

**Figure 2:**
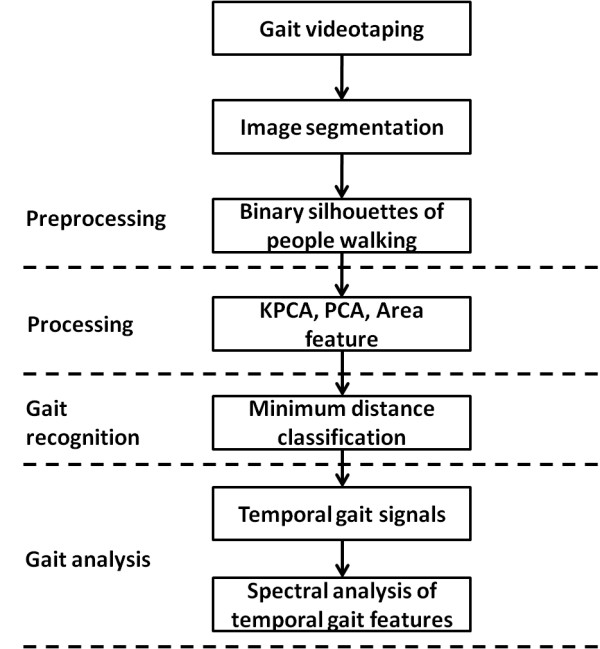
**A flow-chart of gait analysis and recognition**.

The experimental setup and flowchart of the data analyses are illustrated in Figure 1 and 2, respectively. All trials were videotaped using a sampling rate of 15 image frames per second and an image size of 320 × 240 pixels. The spatial resolution was approximately 1.06 pixel/cm. The video files were segmented and separated into sequential images. Using the method and equations presented in binary silhouettes collection, the background of the sequential images can be removed, and the processed sequential images can be transformed into binary images that represent the walking silhouettes to facilitate image noise reduction. Each sequential binary image is then transformed into an encoded one-dimensional matrix containing the biometric features of the gait. KPCA is then applied to detect the biometric features. Using KPCA, the walking silhouettes can be decomposed, and the biometric features of the people walking can be extracted for gait analysis. The efficiency of the KPCA-based feature approach was compared with other competing methods, the area [23] feature approach and the principal component analyses (PCA) [20,24]-based feature approach. To this end, the minimum distance classifier (MDC) [25,26], a numerical approach, was used to classify the different gaits and determine the classification accuracy. The discrete Fourier transform (DFT), which is used to transform a signal in the time domain into a representation in the frequency domain, was applied to transform the coefficients of the KPCA components to understand the spectral power distribution of the different gaits. The KPCA, MDC, and DFT algorithms and the data analysis were implemented using MATLAB (MathWorks, Natick, MA) on a windows XP personal computer equipped with an Intel core 2 duo 6600 processor and 3 GB RAM. The details of implementing the KPCA, MDC, and DFT algorithms for parkinsonian gait analysis are described in KPCA-based feature extraction and heel strike determination, minimum distance classifier (MDC) for classification, and spectral analyses for temporal gait feature signals, respectively. In our preliminary experiments, the KPCA-based feature approach was tested on six healthy adults and compared to the GAITRite^® ^system (CIR Systems Inc., Clifton, NJ, U.S.A) for validation. The preliminary results showed that there was no difference in assessing the kinematic gait parameters of interest, indicating that the KPCA-based feature approach was an easy-to-use and inexpensive tool for measuring the selected kinematic gait parameters. We were convinced of the validity of the KPCA-based feature approach in assessing the gait performance of adults and continued to use it to assess the kinematic gait parameters of the participants in the present study. Detailed descriptions for the setup of the preliminary experiment and the translation of the results are presented in Appendix 1.

### Background Construction

Because we were interested in the silhouette data of a participant walking, the invariant background scene was unnecessary for further gait analyses and should be discarded. The intensity median value of each pixel, which is at the same location through an entire gait sequence, was utilized to construct the background image and is represented as the following:

(1) $$B\left(i,j\right)=\text{media}{\text{n}}_{TN}\left(I_{t}\left(i,j\right)\right)$$

where **I***_t_*(*i, j*) is the brightness at location (*i, j*) in the specific image that corresponds to time instant *t*. **TN **is the total number of images in the entire sequence, and **B**(*i, j*) is the background pixel value.

### Binary silhouette collection

**Figure 3:**
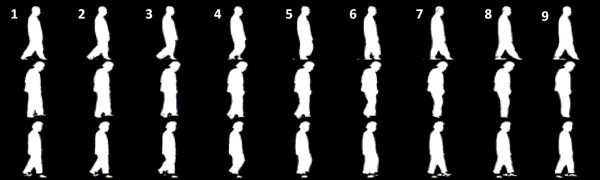
**The trimmed 64 × 64 pixel binary walking sequence silhouettes of non-PD control (top) and Parkinson's disease (PD) patients in the "Drug-Off" (middle) and "Drug-On" (bottom) states**.

To separate the silhouettes from the image frames of the patient walking, the background images for each videotaped gait sequence are prepared using equation (1). A silhouette pixel of the patient walking is acquired by the difference method from [27], represented as equation (2).

(2) $$F_{t}\left(i,j\right)=1-\frac{2\times \sqrt{\left(I_{t}\left(i,j\right)+1\right)\left(B\left(i,j\right)+1\right)}}{\left(I_{t}\left(i,j\right)+1\right)+\left(B\left(i,j\right)+1\right)}\times \frac{2\times \sqrt{\left(256-I_{t}\left(i,j\right)\right)\left(256-B\left(i,j\right)\right)}}{\left(256-I_{t}\left(i,j\right)\right)+\left(256-B\left(i,j\right)\right)}$$

(3) $$\mathrm{THR}=\frac{1}{M}\sum \frac{B\left(i,j\right)}{256}$$

(4) $$\left\{\begin{array}{c} F_{t}\left(i,j\right)=1,\;if\;F_{t}\left(i,j\right)>\mathrm{THR}\\ F_{t}\left(i,j\right)=0,\;if\;F_{t}\left(i,j\right)<\mathrm{THR} \end{array}\right)$$

where **I***_t_*(*i, j*) is the brightness intensity of a pixel (*i, j*) in a particular image frame at an instant *t*, **B**(*i, j*) is the brightness intensity of a prepared background image pixel, ***M ***is the total number of the pixels within the prepared background image (in this case, ***M = ***320 × 240) and ***THR ***is the threshold used to separate a walking silhouette from the original gait sequential image frame. According to equation (3), the brightness levels of all of the prepared background image pixels is averaged and normalized to determine the threshold ***THR***, ranging from [0, 1]. According to equation (4), if **F***_t_(i, j)*, the brightness level of a silhouette pixel, >***THR***, **F***_t_(i, j) *= 1; if **F***_t_(i, j) *<***THR***, **F***_t_(i, j) *= 0. After the binarized procedure, 320 × 240 pixel binary silhouette image frames are acquired. The 320 × 240 pixel binary silhouette image frames are then trimmed to 64 × 64 pixels to preserve the walking silhouette, eliminate redundancies, and reduce the computational costs during image analyses. The trimmed binary silhouettes of the sequential walking image frames from the non-PD control and PD patient in the "Drug-Off" and "Drug-On" states are illustrated in the top region and middle and bottom regions in Figure 3, respectively.

### KPCA-based feature extraction and heel strike determination

Dimensionality reduction and feature extraction approaches have been widely utilized in image processing, such as computing the foreground object areas by counting the number of pixels [23] and PCA [20,24]. In PCA, the representative structure of the principal components that are related to the input variables is an orthogonal transformation of the coordinate system. However, many data types implicitly contain non-linear structures and principal variable components, which are nonlinear and related to the input variables. KPCA is an extension of PCA that uses kernel methods [28] to extract the nonlinear components. In recent years, KPCA has been suggested for various image-processing tasks, such as image noise reduction and compression, as PCA is used to decompose linear combinations of data sets and does not reflect the generation process of natural images [29]. The following is a brief introduction to KPCA.

Consider space *X *with a set of *N *vectors, *x*_1_, *x*_2 _. . ., *x_N_*, which encompasses a set of an *N*-dimensional vector (in this case, *N *= 64 × 64 = 4096 from each trimmed binary silhouette). To analyze the nonlinear components of *X*, the covariance matrix *C *that contains the nonlinear principal components can be acquired by mapping *X *to a feature space, *H*. The mapping equation (5) is shown below.

(5) $$C=\frac{1}{N}\sum_{j=1}^{N}\tilde{\varphi }\left(x_{j}\right)\tilde{\varphi }{\left(x_{j}\right)}^{T}$$

(6) $$\tilde{\phi }\left(x_{i}\right)=\phi \left(x_{i}\right)-m^{\phi }$$

(7) $$m^{\phi }=\frac{1}{N}\overset{N}{\underset{i=1}{\sum }}\phi \left(x_{i}\right)$$

where *ϕ*(*x_i_*) is a nonlinear polynomial function that maps the vectors to *H*, *x_j _*is the *j*-th vector, *N *is the total number of vectors, *T *contains the centralized mapped data of the transposed matrix, *m^ϕ ^*is the mean, and $\tilde{\phi }\left(x_{i}\right)$is the centralized mapped data with *m^ϕ^*. To acquire the eigenvector, *v*, and eigenvalue, λ, of the covariance matrix, *C*, the following equation must be solved:

(8) λv=Cv

Because $\tilde{\varphi }\left(x_{i}\right)$ is a vector with approximately infinite dimensionality, it is difficult to solve the covariance matrix, *C*. Therefore, a new *N *× *N *centralized kernel matrix, $\tilde{K}$, is defined to acquire the eigenvector and eigenvalue of *C*.

(9) $$\tilde{K}=\tilde{\varphi }\left(x_{i}\right)\cdot \tilde{\varphi }\left(x_{j}\right)=K-l_{N}K-Kl_{N}+l_{N}Kl$$

(10) $$K_{ij}=\phi \left(x_{i}\right)\cdot \phi \left(x_{j}\right)=k\left(x_{i},x_{j}\right)$$

(11) $$k\left(x_{i},x_{j}\right)={\left(x_{i}\cdot x_{j}\right)}^{d},\;d>1$$

(12) $${\left(l_{N}\right)}_{ij}=\frac{1}{N}$$

where *i *and *j *are the indices of the row and column, respectively, of vector *x*, *k*(*x_i_*, *x*_j_) is a polynomial kernel function for acquiring the dot product of the vectors from the original space and *d *> 1 because the result of KPCA is identical to that obtained from PCA, where *d *= 1. The relationship between the eigenvector,  ṽ, of the kernel matrix, $\tilde{K}$, and the eigenvector, *v*, of the covariance matrix, *C*, can be expressed as

(13) $$v_{k}=\frac{1}{\sqrt{{\tilde{\lambda }}_{k}}}Q{ṽ}_{k},\;k=1,\text{ 2},\;\cdots \text{,m }$$

(14) $$Q=\left[\tilde{\varphi }\left(x_{1}\right)\;\tilde{\varphi }\left(x_{2}\right)\;\cdots \;\tilde{\varphi }\left(x_{N}\right)\right]$$

where ${\tilde{\lambda }}_{k}$ is a nonzero set of eigenvalues of  ṽ, *m *is the number of non-zero eigenvalues and  Q is a centralized mapped data set. After projecting *Q *to the feature space constructed by *v*_1_, *v*_2_,..., *v_m_*, the *k*^th ^KPCA feature vector, *y_k_*, can be represented as

(15) $$y_{k}=v_{k}^{T}Q=\frac{1}{\sqrt{{\tilde{\lambda }}_{k}}}{ṽ}_{k}^{T}Q^{T}Q=\frac{1}{\sqrt{{\tilde{\lambda }}_{k}}}{ṽ}_{k}^{T}\tilde{K}$$

where *T *refers to the transposed matrix.

To summarize, the KPCA computation can be separated into three steps. The first step is to determine parameter *d *in (11) for the polynomial kernel function and derive kernel matrix $\tilde{K}$ according to equation (9). The second step is to derive the *k*^th ^major eigenvector, ${ṽ}_{k}$, of the *N × N *centralized kernel matrix, $\tilde{K}$, and acquire the *k*^th ^coordinate vector, *v_k_*, using equation (13). The third step is to project the centralized mapped data set, *Q*, to the feature space using equation (15) and to acquire the *k*^th ^KPCA feature vector, *y_k_*.

**Figure 4:**
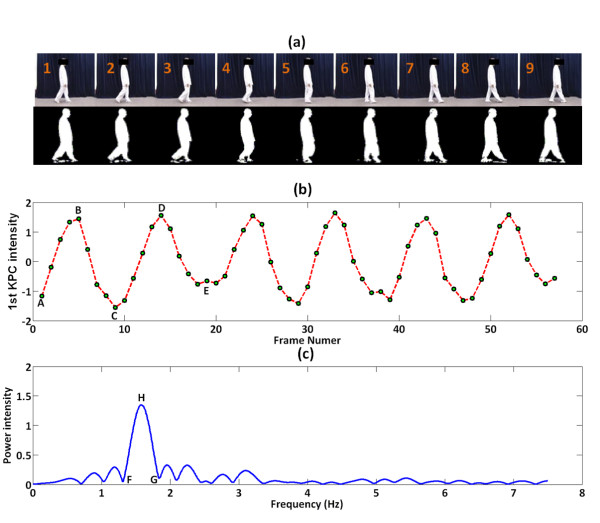
**An example of step image frames from a non-PD control subject. (a)** Step image frames from a non-PD control subject. The top panel is the original sequential walking image frames with 240 × 320 pixels. The bottom panel is the trimmed 64 × 64 pixels binary silhouettes of the top image frames. **(b) **The magnitudes of the associated sequential primary KPCA components. For simplicity, the primary KPCA component is denoted as *^1st^KPC*. The green dots indicate the magnitudes of the sequential *^1st^KPCs*. **(c) **The power spectrum of (b) using a 2048-point DFT and rectangular window with a length, L, of 64 points.

In this study, KPCA is used to reduce the dimensionality of image frames with multiple nonlinear components. The KPCA-based feature approach selects the primary components from a walking image sequence, forming a biometric feature vector to represent a given participant. The sequential gait image frames and the associated sequential primary KPCA components (*^1st^KPC*) of a non-PD control are shown in Figure 4(a) and 4(b). After comparing the gait sequences and associated *^1st^KPC *waveforms, the *^1st^KPC *value (fifth dot, Figure 4(b), **B**) reaches a local maximum value when a participant performs a mid-swing event (***frame 5***, Figure 4(a)). Moreover, a heel strike event (***frame 1***, Figure 4(a)) corresponds to a local minimum *^1st^KPC *value (first dot, Figure 4(b), **A**). Similarly, in Figure 4(b), the *^1st^KPC *values are at local maximums (fifth and thirteenth dot, Figure 4(b), **B **and **D**, respectively) and minimums (first and ninth dot, Figure 4(b), **A **and **C**, respectively) at the moment when the non-PD control participant is performing mid-stances and heel strikes, respectively.

Using the temporal *^1st^KPC *waveforms, the moment of occurrence of the heel strike can be determined. The distance between two heel strikes can be estimated by the two locations of the heel in the binary 240 × 320 pixels image frames. As a result, the kinematic gait parameters, which depend on the time period and distance between two heel strikes that are performed with the same foot, gait cycle time, stride length, walking velocity and cadence can be estimated. The power spectrum of the temporally associated *^1st^KPC *is plotted in Figure 4(c), and the main lobe frequency reflecting the step frequency of the non-PD control is located at approximately 1.67 Hz.

### Minimum Distance Classifier (MDC) for classification

The MDC [25,26] is a numerical approach used for classify unknown data to classes which minimize the distance between the data and the class in multi-feature space. Because the distance is defined as an index of similarity, the minimum distance is identical to the maximum similarity. In the current study, the efficiency of area, PCA-based, and KPCA-based feature approaches are compared by evaluating their classification accuracy. An area feature vector is obtained by counting the number of pixels of a particular participant's walking image sequence. On the other hand, the primary PCA and KPCA components per frame are used to form the PCA and KPCA feature vectors for a particular participant, respectively. Gait patterns are classified with a MDC, which is sufficient for the evaluation of feature efficiencies in this study. MDC is used in this study to classify a feature vector *y *to the to *i*th class *I *whose mean m_*i *_has a minimum Euclidian distance to *y*. The minimum distance classifier can be expressed as

(16) $$I=\arg\underset{i}{\min}\left\{{\left(y-m_{i}\right)}^{T}\left(y-m_{i}\right)\right\}$$

where (***y ***- **m***_i_*)*^T ^*(***y ***- **m***_i_*) is the Euclidian distance; *T *is the data of the transposed matrix.

### Spectral analyses for temporal gait feature signals

The DFT is a specific method to transform a function in the time domain into the frequency domain for understanding the spectral power distribution of the function [30]. For the spectral gait analyses, *^1st^KPC *is transformed with DFT. The main lobe frequency (corresponding to **H **in Figure 4(c)) of the *^1st^KPC *spectrum, representing the gait parameter of the step frequency for a participant, is then computed. Afterwards, the sums of the powers within the main lobe between **F **and **G **in Figure 4(c) is calculated. The sums of the powers within the main lobe are denoted as E_M, N_, E_M, OFF _and E_M, ON _for a non-PD control, a "Drug-Off" PD patient and a "Drug-On" PD patient, respectively. The low- and high-frequency counterparts for a participant, denoted as E_L, N_, E_L, OFF _and E_L, ON _and E_H, N_, E_H, OFF _and E_H, ON_, respectively, are also similarly calculated.

### Statistical analysis

The study was designed to identify the gaits and quantify the gait parameters of the non-PD controls and the "Drug-On" (proper L-dopa treatment received one hour later) and "Drug-Off" (at least 12 hours after the withdrawal of L-dopa) PD patients. A paired *t*-test was used to examine the differences among non-PD controls, "Drug-Off" and "Drug-On" PD patients. To examine whether the BMI and age make impacts on recognizing the non-PD control and "Drug-Off" gaits, an analysis of covariance (ANCOVA) test for measurements with covariates BMI and age was applied to evaluate the differences in gait parameters between the two groups. A value of *p *less than 0.05 is considered to be statistically significant. A least significant differences LSD *post hoc *test is performed.

## Results

### UPDRS part III subscores of the PD patients in the "Drug Off" and "Drug On" states

Five subscores--axial score (summation of UPDRS items 18, 22 with neck only, 27, 28, 29, and 30), limb akinesia (summation of items 23, 24, 25, and 26), limb rigidity (item 22 with neck excluded), limb tremor (summation of items 20 and 21), and part III (summation of items 18 to 31)--are computed according to the UPDRS part III motor scores (Table 2) to describe the motor deviation of PD patients in different states (Table 3). After the L-dopa treatment, the five subscores of all PD patients are improved; 10 PD patients show an increase of less than 20 points on the Part III scores.

**Table 2:** UPDRS part III motor scores during the"Drug-Off" and"Drug-On" states.

Patient	1	2	3	4	5	6	7	8	9	10	11	12
**Drug**	**Off/On**	**Off/On**	**Off/On**	**Off/On**	**Off/On**	**Off/On**	**Off/On**	**Off/On**	**Off/On**	**Off/On**	**Off/On**	**Off/On**

*18. speech*	2/2	1/1	2/2	0/0	1/1	0/0	1/1	1/1	1/0	1/1	1/1	1/1
*19. Facial expression*	1/1	0/0	2/2	1/1	1/0	0/0	2/1	1/1	2/1	1/1	1/1	1/1
*20. Tremor at rest*												
*Face*	0/0	0/0	0/0	0/0	0/0	0/0	0/0	0/0	0/0	0/0	0/0	0/0
*Hand R, L*	1,0/1,0	3,1/1,0	0,3/0,3	2,1/2,0	0,0/0,0	1,0/1,0	1,1/0,0	2,0/0,0	0,1/0,0	0,3/0,3	0,0/0,0	0,0/0,0
*Feet R, L*	1,2/0,0	1,0/0,0	2,0/1,1	1,0/0,0	0,0/0,0	0,0/0,0	0,0/0,0	2,0/0,0	1,0/0,0	1,2/1,2	0,0/0,0	0,0/0,0
*21. Action tremor R, L*	0,0/0,0	2,1/1,0	0,0/0,0	1,1/0,0	0,0/0,0	1,0/1,0	1,1/1,1	1,1/0,0	2,2/0,0	0,3/0,2	0,0/0,0	0,0/0,0
*22. Rigidity *												
*Neck*	2/1	2/2	2/2	2/2	0/0	0/0	2/1	2/1	2/0	1/0	1/1	2/2
*UE R, L*	2,1/1,0	3,2/2,1	2,3/1,2	2,2/2,1	1,2/0,2	3,2/2,1	3,2/2,1	1,2/0,1	1,2/0,0	0,2/0,1	1,3/0,2	2,2/2,2
*LE R, L*	2,1/1,1	2,2/2,1	3,3/2,3	2,3/1,2	0,1/0,1	1,0/0,0	2,2/1,1	2,2/0,1	3,2/2,1	0,1/0,0	1,2/1,2	2,1/2,1
*23. Finger taps R, L*	0,2/0,0	2,1/1,1	2,2/1,2	1,1/0,1	1,2/1,1	1,1/1,0	2,3/1,1	2,2/1,1	1,2/1,2	0,3/0,2	1,2/1,1	1,2/1,2
*24. Hand grips R, L*	0,1/0,0	2,1/1,1	1,2/1,2	2,1/1,1	0,1/0,1	1,1/1,1	2,2/1,1	2,1/1,1	2,2/1,2	0,2/0,2	1,3/1,2	1,1/1,1
*25. RAMH R, L*	0,2/0,2	3,1/1,0	2,3/1,3	2,2/2,2	1,2/0,0	1,0/1,0	2,2/1,1	2,2/2,2	1,2/1,1	0,3/0,2	1,3/1,3	1,2/1,1
*26. Leg agility R, L*	2,2/0,0	2,1/1,0	3,3/3,3	1,1/0,1	0,1/0,1	1,0/0,0	1,1/0,0	1,1/0,0	2,2/0,0	0,1/0,1	1,2/1,1	0,0/0,0
*27. Arise from chair*	3/0	2/1	2/1	1/0	0/0	1/0	1/0	1/1	1/0	0/0	1/1	0/0
*28. Posture*	1/1	1/1	1/1	1/1	1/1	1/1	3/2	2/2	1/1	0/0	1/1	0/0
*29. Gait*	1/0	1/1	2/2	1/0	1/0	1/0	1/1	2/1	2/0	1/1	1/1	0/0
*30. Postural stability*	2/2	0/0	1/1	2/2	1/1	1/1	2/1	2/2	2/1	1/1	1/1	0/0
*31. Body bradykinesia*	2/1	2/1	2/2	1/1	1/0	1/1	2/0	2/1	2/1	2/2	2/2	1/1
*Part III: total*	33/14	39/21	48/42	35/23	18/10	19/12	42/20	39/20	41/14	28/22	30/25	20/19

R: right; L: left; UE: upper extremity; LE: lower extremity; RAMH: rapid alternating movements of hands.

**Table 3:** Definition of the five subscores.

Subscores	UPDRS part III motor items
*Axial score*	18, 22 (neck only), 27, 28, 29, 30
*Limb akinesia*	23, 24, 25, 26
*Limb rigidity*	22(without neck)
*Limb Tremor*	20, 21
*Part III*	18 - 31

### Comparison of the efficiencies of the approaches

Classification accuracies using MDC based on the area feature, PCA-based and KPCA-based features to identify different gaits are presented by confusion matrices in Table 4. In identifying the non-PD controls from the "Drug-Off" and "Drug-On" PD patients, the separation capabilities of area, PCA-based and KPCA-based features are similar. However, area feature is inadequate in identifying "Drug-On" PD patients. Only one of them is classified correctly, whereas the others are mis-classified as non-PD controls or "Drug-Off" PD patients. On the other hand, results from the KPCA-based feature approach provide an average accuracy rate of 80.51%.

**Table 4:** The confusion matrices use the area feature, PCA and KPCA approaches to classify different gaits.

	Area feature			PCA			KPCA		
			
	Non-PD	Drug-Off	Drug-On	Non-PD	Drug-Off	Drug-On	Non-PD	Drug-Off	Drug-On
Non-PD	9	0	3	10	1	1	10	0	2
Drug-Off	1	8	3	2	7	3	2	10	0
Drug-On	6	5	1	3	1	8	2	1	9
Predicted/actual	9/12	8/12	1/12	10/12	7/12	8/12	10/12	10/12	9/12
Accuracy	75%	66.67%	8.33%	83.33%	58.33%	66.67%	83.33%	83.33%	75%
Average accuracy	50.16%			69.44%			80.51%		

### Kinematic gait parameters among the different groups

The KPCA-based method was used to extract the gait parameters from the sequential gait video frames from the non-PD controls, "Drug-Off" PD patients and "Drug-Off" PD patients. The average gait cycle times (s), stride lengths (cm), walking velocities (cm/s), and cadences (steps/min) of these groups are presented in Table 5. Although the PD patients showed improvements in all gait parameters after receiving medical treatment, only stride length showed significant improvement. Compared to the "Drug-Off" PD patients, the non-PD controls manifested better gait performance in terms of stride length and walking velocity, but their performance was worse than the "Drug-On" PD patients across all kinematic gait parameters. Moreover, the non-PD controls showed no significant differences in the kinematic gait parameters compared to the "Drug-Off" and "Drug-On" PD patients. ANCOVA measurements were used to analyze the interaction between factors (BMI and age) and groups (Non-PD and Drug-Off). We find found that there were no significant differences in the interactions between the factors and groups.

**Table 5:** Kinematic and spectral gait parameters (Mean ± SD).

	Non-PD (n = 12)	Drug-Off (n = 12)	Drug-On (n = 12)	BMI	AGE
Gait cycle time (s)	1.21 ± 0.08	1.16 ± 0.15	1.15 ± 0.12	N.S	N.S
Stride length (cm)	105.48 ± 6.95	85.54 ± 17.21	^ξ^105.94 ± 22.90	N.S	N.S
Walking velocity (cm/s)	87.35 ± 8.45	76.35 ± 22.84	93.68 ± 23.35	N.S	N.S
Cadence (steps/min)	99.33 ± 6.04	106.45 ± 15.09	105.88 ± 12.33	N.S	N.S
Step frequency (Hz)	1.66 ± 0.11	1.48 ± 0.34	^ξ^1.74 ± 0.18	N.S	N.S
EL (%)	9.55 ± 7.84	16.21 ± 9.60	^ξ^9.40 ± 4.56	N.S	N.S
EM (%)	*75.07 ± 9.36	63.89 ± 11.63	72.58 ± 8.63	N.S	N.S
EH (%)	15.37 ± 3.10	19.88 ± 8.25	18.00 ± 6.31	N.S	N.S

*, ^ξ ^are significant (*p *< 0.05) regarding the non-PD control and PD patients in the "Drug-Off" state and PD patients in the "Drug-Off" and "Drug-On" states. EL, EM, and EH are the percent of energy distributed in the low-frequency band, main-frequency band, and high-frequency band, respectively. Interactions between *factors *(BMI and AGE) and *Groups *(Non-PD and Drug-Off) were assessed by ANCOVA. N.S is non-significant (*p >*0.05).

**Figure 5:**
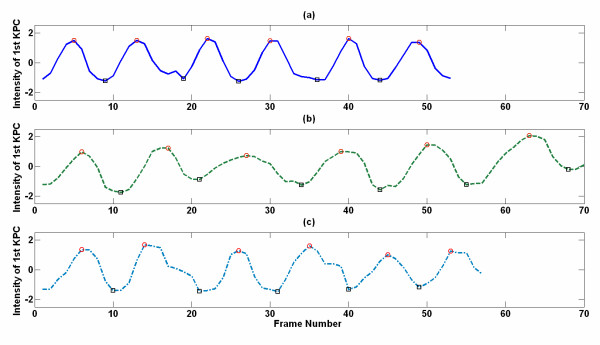
**The representations of 1^st^KPC gait feature for non-PD control subjects and PD patients, respectively. (a)** The *^1st^KPC *waveform of a selected non-PD control participant. **(b) **The *^1st^KPC *waveform of a selected PD patient in the "Drug-Off" state. **(c) **The *^1st^KPC *waveform of the same PD patient in the "Drug-On" state. The red circles and black squares represent the local maximums and minimums, which reflect the occurrences of mid-swings and heel strikes, respectively.

We presents examples of the *^1st^KPC *waveforms from the non-PD Controls, "Drug-Off" PD patients and "Drug-On" PD patients in Figure 5. Interestingly, the *^1st^KPC *waveforms of the "Drug-On" PD patients were similar to those of the non-PD controls, whereas the waveforms of the "Drug-Off" PD patients tended to be irregular.

### Power spectrum of temporal gait signals

**Figure 6:**
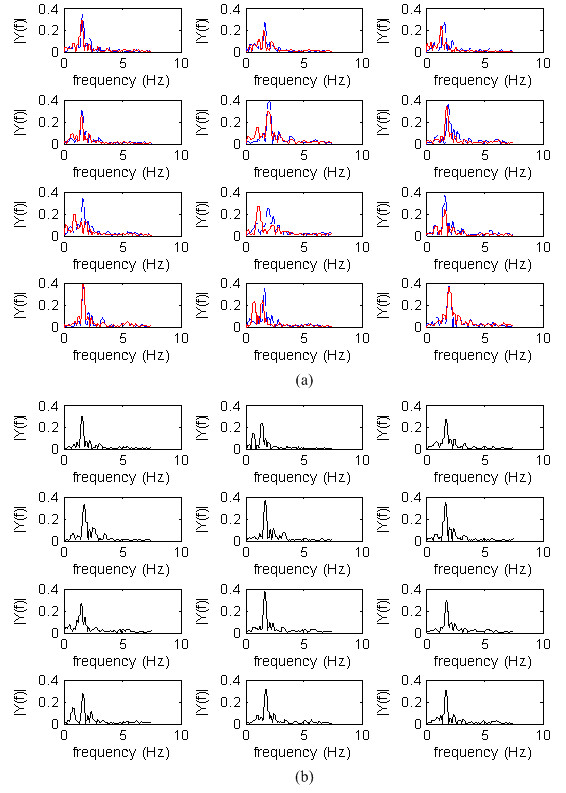
**The gait frequency spectra of (a) PD patients in the "Drug-Off" state (red solid line) and the "Drug-On" state and (b) the non-PD controls**.

The gait frequency spectra of the PD patients in the "Drug-Off/On" states and the non-PD controls are shown in Figure 6. The average step frequencies of the non-PD controls and PD patients in the "Drug-On" and "Drug-Off" states are 1.661, 1.743 and 1.543 Hz, respectively. The comparisons of the step frequency and spectrum power distributions among the three groups are shown in Table 5. The results from a paired *t*-test indicate that "Drug-On" PD patients show significant improvement in step frequency. Moreover, E_M, NON _is significantly larger than E_M, OFF_, and E_L, ON _is significantly larger than E_L, OFF_.

## Discussion

### The accuracy of the KPCA-based gait recognition method

As the parkinsonian gait is commonly accompanied by not only slowness in walking and shuffling steps, but also a reduction in hand swing and a stooped posture, thus, information regarding upper extremities and trunk movements can be used in identifying a PD patient [29]. The silhouette approach proposed in this study aims to develop a parkinsonian gait recognition method. Two parkinsonian gait recognition algorithms, the KPCA-based and PCA-based, as well as the area feature approaches, were tested for their abilities to identify and classify the gait of "non-PD" controls and the PD patients in different states. The area feature approach is worst at identifying the "Drug-On" PD patients. Only one out of 12 PD patients in the "Drug-On" state is correctly identified and classified; the rest are incorrectly identified as non-PD controls or "Drug-Off" PD patients. The area feature approach calculates the area occupied by an object (in this case, a participant) in an image frame (i.e., the number of pixels), which may be similar under different conditions. Thus, the poor accurate rate of the area feature approach is not surprising. The average accurate rate of the PCA-based feature approach is 69.44%, which is better than the area feature method. Nonetheless, the average accuracy rate of the KPCA-based feature approach is the highest at 80.51%. Such results are expected; although PCA is appropriate in decomposing images with linear components, the spatiotemporal characteristics associated with abnormalities in gait and upper extremity and trunk movements contain nonlinear principal components.

### Gait parameter analysis

Quantitative gait performance is an important reference for monitoring the progression of Parkinson's disease and the improvement brought about by scheduled treatments. Our results show that PD patients who receive the L-dopa treatment show improvement in their gait cycle time, stride length, walking velocity, cadence, and step frequency. Such results are consistent with the findings in prior studies [31-33]. However, the PD patients show significant improvements only in their stride length and step frequency in the "Drug-On" state, whereas the improvements in gait cycle time, cadence, and walking velocity are not significant.

It has been reported that PD patients with advanced motor symptoms exhibit significantly improved stride length and walking speed and slightly increased cadence after the L-dopa treatment [33-36]. However, the results of the present study showed that the L-dopa treatment slightly increased cadence but significantly improved only the stride length. Due to the relatively small number of subjects and the absence of PD patients with advanced motor symptoms, the improvement brought about by the L-dopa treatment was not robust. Thus, the PD patients in the "Drug-On" state exhibited few significant differences in gait parameters, although reductions in gait irregularity and motor symptoms were observed.

Moreover, the differences in the gait parameters between the PD patients in the "Drug-On" state and the "non-PD" controls were not significant. There are two possible explanations for this seemingly counterintuitive result. According to Table 2, 9 PD patients scored less than 40 points on part III in the "Drug-Off" state, and the part III scores of 11 PD patients improved to less than 25 points in the "Drug-On" state. These results once again indicate that most of the PD patients in this study are in the mild stages of the disease and present no significant deficits or deterioration in motor functions; they move normally or with only a slight impairment after receiving L-dopa. Although the non-PD controls recruited were healthy adults of nearly the same age as the patients, when the ANCOVA model was applied with age as a covariate, the statistical outcomes were identical to those in a model without including age. Given that the healthy and patient groups had similar physical conditions, it is not surprising that there were no significant differences in gait performance between the two groups. The non-PD controls and PD patients had similar BMIs, but the standard deviation in the PD group was higher. Despite the heterogeneity in the BMIs, ANOCVA revealed that the BMI did not affect the gait parameters in the present case.

As shown in Figure 5, the *^1st^KPC *waveforms from walking cycles performed by a "non-PD" controls and a PD patient during the "Drug On" state are similar. Although this finding indicates that the gait patterns of "Drug-On" PD patients is similar to those of healthy adults, a longer gait cycle time and the freezing of gait and slowness in motion resulted from body bradykinesia can be identified from the irregular and gentle parts presented in Figure 5(c). Due to the slow and asymmetry and unsTable walking of the PD patients during the "Drug-Off" state, the *^1st^KPC *waveforms appear to be irregular and different from the non-PD control subject's *^1st^KPC *waveform. In fact, the slowness in the walking cycle shifts the main frequency of the *^1st^KPC *waveform toward a lower frequency band. Compared with the PD patients during the "Drug-On" state, approximately 8% of the energy is shifted from the main frequency (Drug On: 73.86%, Drug Off: 66.10%) to a lower frequency band (Drug On: 8.34%, Drug Off: 16.95%) for the "Drug-Off" PD patients. The change in the power distribution causes the temporal *^1st^KPC *waveform, resulting in a failure to form regular waveforms resembling those of non-PD controls. Such results show that the lack of dopamine in the basal ganglia circuit in the brain may cause abnormalities and irregularities in the gait profile.

### The limitations of the proposed approach

There are some limitations of the present study. First, because only a digital camera was used to capture the lateral view of the walking silhouettes, the proposed method provides no information useful for examining the gait asymmetry that is known to be an important factor for monitoring the progression of PD. An additional camera to capture the frontal view and an algorithm to discriminate the left and right legs may allow the proposed method to detect gait asymmetry. Second, the proposed method lacks the ability to perform kinetic gait analysis. Kinetics permits computation of the net forces or net moments of the force at each joint at every stage of the gait cycle. This greatly helps researchers to determine the activity and contribution of individual muscles. The use of electromyography and acceleration measurements in motions during the gait cycle will provide kinetic gait analysis and will enhance the proposed method.

Despite the mentioned shortcomings, the proposed KPCA-based feature approach that shares identical requirements for computation, storage, and equipment with the existing silhouette methods provides not only recognition of but also quantification of parkinsonian gait to aid in clinical diagnoses and evaluation of the disease's progression. In addition, the proposed approach provides the power spectrum of the participant's gait as additional information to analyze the irregularity of the actions of PD patients.

## Conclusion

The ability to evaluate treatments for Parkinson's disease is an important issue. However, previous research has been hindered due to the lack of a tool that can be easily installed, provide prompt gait analysis, facilitate data collection and gait analysis, and lower a patient's level of exertion during the examinations. In this study, a computer vision-based gait analysis approach that is different from other sensor- or marker-based approaches is developed. The proposed method uses kernel-based principal component analysis; it only requires a digital camera and a decorated corridor to facilitate the classification and quantification of specific gait patterns. Although there are few significant differences among the gait patterns, the proposed method presents encouraging classification accuracy rates of 80.51% in identifying different gaits. This technique provides a practicable reference for clinicians and researchers with which to obtain the quantitative gait parameters and assess the progression of Parkinson's disease in the motor section of the brain using ambulation patterns recorded in monocular image frames.

## Abbreviations

PD: Parkinson's disease; KPCA: kernel-based principal component analysis; UPDRS: Unified Parkinson's Disease Rating Scale; L-dopa: levodopa; MDC: minimum distance classifier; DFT: discrete Fourier transform; PCA: principal component analysis; *^1st^KPC: *is the magnitude of the first principal component; LSD: least significant difference.

## Competing interests

The authors declare that they have no competing interests.

## Authors' contributions

SWC and LDL carried out the concept and design of the study, and interpreted the data. SWC performed the statistical analysis. SHL and HYL participated in the acquisition of gait data. SWC, LDL, SHL, and ST drafted the manuscript. TSK, CTL, YCP, LRW, and YYC provided critical revision of the manuscript for important intellectual content. SHL and YYC conceived of the study, and obtained funding. TSK, CTL, and YYC provided administrative, technical, and material support for the study. YYC supervised the study. Also, all authors read and approved the final manuscript.

## Appendix 1

### Comparing the KPCA-based feature approach with the GAITRite^® ^method for validation

To evaluate the validity of the proposed KPCA-based method in quantifying gait performance to aid clinical diagnoses and further applications, the quantitative gait parameters from the proposed approach were compared with the outcomes of the GAITRite^® ^system (CIR Systems Inc., Clifton, NJ, U.S.A) prior to the actual experiment.

The GAITRite^® ^system is an instrumented walkway system that has been validated as a reliable tool for the measurement of kinematic gait parameters [37]. Six healthy male volunteers with an average age of 54 years (max = 76.5 years, min = 46 years, S.D. = 9.7 years) were recruited, and they provided informed consent for participation. Using an identical experimental setup to that employed in the present study, these volunteers were asked to perform four walking trials at their natural pace, and a total of 24 trials were collected.

**Figure 7:**
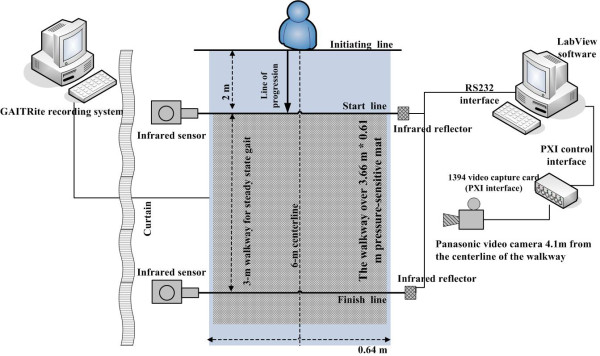
**Equipment setup used to measure gait parameters with the GAITRite^® ^mat and its recording system, and the KPCA-based method**.

The equipment setup used to perform the concurrent gait analysis by the GAITRite^®^- and KPCA-based methods are shown in Figure 7. Gait analysis was performed using an electrical spatial and temporal analysis system (GAITRite^® ^system). The GAITRite^® ^system is a 4.6-m-long electronic walkway that connects to the serial port (19,200 baud rate) of a windows XP computer. The walkway is 1/8 inch thick and contains 16,128 sensors sandwiched between a thin vinyl cover on top and a rubber bottom. The active senor area is 0.61 m wide by 3.66 m long. GAITRite^® ^software (ver. 3.8) was used to process the footstep data and to provide quantified temporal and spatial parameters.

The Panasonic video camera (model PV-GS400, Japan) used to videotape the walking trials was mounted on a tripod and positioned midway between the start and finish lines of the walkway, with the camera's field of view perpendicular to the long axis of the walkway. All trials were videotaped using a sampling rate of 15 image frames per second and an image size of 320 × 240 pixels. The plane of the camera's shutter was located 4.1 m from the centerline of the walkway. The size of the field of view ensured that two pairs of infrared sensors, which were aligned with the start and finish lines, could be seen in the camera's viewfinder. One graphical programming utility was developed using the NI LabVIEW environment (LabVIEW 8.5, National Instruments, Austin, TX, U.S.A) and a 1394 video capture card (Model PXI-8252, National Instruments, Austin, TX, U.S.A) to synchronize the motion detection and gait video capture.

In a preliminary experiment, the participant stood at the initiating line with his toes just behind the line. The participant was asked to walk through a 6-m corridor decorated with a navy curtain, and the 3-m GAITRite^® ^walkway for steady-state gait was defined by two pairs of infrared sensors that were aligned with the start and finish lines. When the infrared beam was broken by the participant's advancing lower leg, the infrared reflector transmitted a TTL logic signal to the LabVIEW utility on the PC side via a serial RS232 port, providing timestamps to determine the timing of the closest playback frame at the start and end of each walking trial.

**Figure 8:**
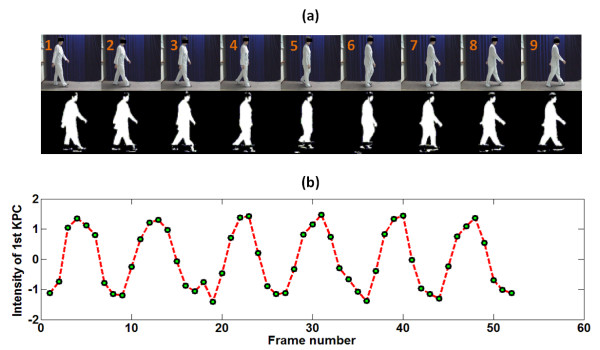
**Sequential gait image frames from a healthy subject walking on the GAITRite® walkway system. (a)** Sequential gait image frames from a healthy subject walking on the GAITRite walkway system. The top panels are the original sequential walking image frames containing 240 × 320 pixels. The bottom panels are the trimmed 64 × 64 pixel binary silhouettes of the top image frames. **(b) **The *^1st^KPC *waveform of a selected non-PD control volunteer participating in the preliminary experiment. The green dots indicate the magnitudes of the sequential *^1st^KPCs*.

The GAITRite^® ^system and the video camera simultaneously collected footstep data during the steady-state walking period. The gait sequence image frame and the *^1st^KPC *waveform of a healthy subject are shown in Figure 8(a) and 8(b), respectively.

Results from the KPCA-based method were acquired using processing procedures identical to those used in the actual experiment. A paired Student's *t*-test was conducted to examine the differences between the gait parameters (gait cycle time, stride length, walking velocity and cadence) measured by the GAITRite^® ^system and the KPCA-based method. According to Table 6, there were no significant differences (*p*-value > 0.05) in the gait cycle time, stride length, walking velocity, or cadence detected using these two methods, indicating that the KPCA-based method and the GAITRite^® ^system yielded comparative gait measurements. This finding was expected because the results of the two methods were also correlated with respect to the spatial measures recorded concurrently during the subject's walking trials. We are hence convinced of the validity of the KPCA-based method for acquiring adult gait cycle time, stride length, walking velocity, and cadence.

**Table 6:** Comparison of gait parameters assessed by the GAITRite^® ^system and the KPCA-based method

Gait Parameters	KPCA	GAITRite^®^	*p-value*
Gait cycle time (s)	1.26 ± 0.13	1.26 ± 0.12	N.S.
Stride Length (cm)	116.81 ± 6.55	116.48 ± 6.68	N.S.
Walking Velocity (cm/sec)	92.95 ± 10.55	92.97 ± 10.59	N.S.
Cadence (steps/min)	95.90 ± 9.99	95.79 ± 9.97	N.S.

N.S is non-significant (*p >*0.05).
